# Evaluating breastfeeding among active Tunisian mothers

**DOI:** 10.1192/j.eurpsy.2025.2428

**Published:** 2025-08-26

**Authors:** A. Hrairi, N. Rmadi, O. Ben Ayed, A. Kchaou, F. Dhouib, E. Belkhir, M. Hajjaji, K. Jmal Hammami

**Affiliations:** 1 Occupational medicine department, Hedi chaker university hospital, University of Sfax; 2 Family medicine department, university of Sfax, Sfax; 3 Lactabelle Society, Tunis, Tunisia

## Abstract

**Introduction:**

Breastfeeding is an important determinant of both mother and child healths regarding its physical and psychological benefits. Working mothers usually consider the work as a barrier to breastfeeding, thus this population needs a particular attention to enhance their breastfeeeding rate.

**Objectives:**

Determining the prevalence of breastfeeding among active women in Tunisia and evaluating their perceptions and their practices towards this process.

**Methods:**

We have conducted a descriptive and cross sectional study. Data were collected during one week through an online questionnaire designed by Google Forms and distributed via Social media. It had included active women who had a child aged between 6 months and 5 years. Exclusive breastfeeding (EBF) is feeding the baby only breast milk (not any other foods or liquids).

**Results:**

A total of 51 active mothers had participated with a mea age of 32.4±3.8 years. A proportion of 47.1% worked in healthcare facilities. Nineteen participants were doctors. All the participants had experienced breastfeeding with a mean duration of 12.7 ±7.5 months [ranging from 2 to 25 months]. The mean duration of EBF was 3.1±2.0 months [ranging from 0 to 6 months]. A percentage of 64.7% had experienced mixed feeding. Regarding breastfeeding perceptions, 92.2% of women always believe that breastmilk was the right option to feed their babies. Tweenty two (43.1%) always believe that they could successfuly breastfeed their child and sixteen (31.4%) consider that they was usually sure that they were able to balance between work and breastfeeding. Participants mothers had returned to their job within a mean of 12.7 ±7.5 months [ranging from 2 to 25 months]. After returning to work, 56.9% of participants consider that breastfeeding represented a big challenge. EBF was negatively associated to the number of working hours /week (p=0.026).

**Conclusions:**

The participation of women in the labor force makes the continuation of breastfeeding a challenging option. Thus, promoting and supporting breastfeeding in the workplace are essential and have the potential to ameliorate babies and mothers lives, and to positively affect the employees productivity and well-being.

**Disclosure of Interest:**

None Declared

